# Retraction Note: Potential biological applications of environment friendly synthesized iron oxide nanoparticles using *Sageretia thea* root extract

**DOI:** 10.1038/s41598-025-31528-7

**Published:** 2025-12-09

**Authors:** Muhammad Israeel, Javed Iqbal, Banzeer Ahsan Abbasi, Shumaila Ijaz, Rafi Ullah, Farishta Zarshan, Tabassum Yaseen, Gul Khan, Ghulam Murtaza, Iftikhar Ali, Khaloud Mohammed Alarjani, Mohamed S Elshikh, Muhammad Rizwan, Shoaib Khan, Rashid Iqbal

**Affiliations:** 1 Department of Botany, Bacha Khan University, Charsadda, 24420 Khyber Pakhtunkhwa Pakistan; 2 Department of Botany, Rawalpindi Women University, 6th Road, Satellite Town, Rawalpindi, 46300 Pakistan; 3 School of Biomedical Engineering, Shenzhen University Medical School, Shenzhen University, Shenzhen, 518060 China; 4 Faculty of Transport and Aviation Engineering, Silesian University of Technology, Katowice, 40-019 Poland; 5 School of Agriculture, Yunnan University, Kunming, 650504 Yunnan China; 6 Center for Plant Sciences and Biodiversity, University of Swat, Charbagh, 19201 Pakistan; 7 Department of Genetics and Development, Columbia University Irving Medical Center, New York, NY USA; 8 Department of Botany and Microbiology, College of Science, King Saud University, Riyadh, 11451 Saudi Arabia; 9 Institute of Crop Science and Resource Conservation (INRES), University of Bonn, 53115 Bonn, Germany; 10 Department of Chemistry, Abbottabad University of Science and Technology (AUST), Abbottabad, 22500 Pakistan; 11 Department of Agronomy, Faculty of Agriculture and Environment, The Islamia University of Bahawalpur, Bahawalpur, 63100 Pakistan; 12 Department of Life Sciences, Western Caspian University, Baku, Azerbaijan

Retraction of: *Scientific Reports* 10.1038/s41598-024-79953-4, published online 16 November 2024

The Editors have retracted this Article.

After publication, concerns were raised about the data presented in this study. Specifically, repeating fragments are observed in the background of a graph depicting XRD pattern in Figure 4. Concerns were also raised regarding the shape of the UV-VIS spectroscopy graph in Figure 2. Additionally, the SEM image presented in Figure 6 appears highly similar to Figure 4 from^1^, where it depicts different conditions.

The Authors could not provide all of the necessary raw data to verify the veracity of the results.

Khaloud Mohammed Alarjani agrees to this retraction. Rashid Iqbal, Mohamed S Elshikh, Javed Iqbal and Banzeer Ahsan Abbasi do not agree to this retraction. Muhammad Israeel, Shumaila Ijaz, Rafi Ullah, Farishta Zarshan, Tabassum Yaseen, Gul Khan, Ghulam Murtaza, Iftikhar Ali, Muhammad Rizwan and Shoaib Khan have not responded to any correspondence from the Editor about this retraction.

## References

[1] Abbasi, B. A. et al. Environmentally friendly green approach for the fabrication of silver oxide nanoparticles: Characterization and diverse biomedical applications. *Microsc. Res. Tech.* **83** (11), 1308–1320 (2020). 32666568 10.1002/jemt.23522

